# The Inflammation-Related Gene S100A12 Is Positively Regulated by C/EBPβ and AP-1 in Pigs

**DOI:** 10.3390/ijms150813802

**Published:** 2014-08-08

**Authors:** Xinyun Li, Juan Tang, Jing Xu, Mengjin Zhu, Jianhua Cao, Ying Liu, Mei Yu, Shuhong Zhao

**Affiliations:** Key Lab of Agricultural Animal Genetics, Breeding and Reproduction of Ministry of Education and Key Laboratory of Swine Genetics and Breeding of Ministry of Agriculture, Huazhong Agricultural University, Wuhan 430070, China; E-Mails: xyli@mail.hzau.edu.cn (X.L.); tangjuan1113@yeah.net (J.T.); hzauxujing@163.com (J.X.); zhumengjin@mail.hzau.edu.cn (M.Z.); jhcao@mail.hzau.edu.cn (J.C.); Liuying_K4@163.com (Y.L.); yumei@mail.hzau.edu.cn (M.Y.).

**Keywords:** pig, S100A12, C/EBPβ, AP-1, transcriptional regulation

## Abstract

S100A12 is involved in the inflammatory response and is considered an important marker for many inflammatory diseases in humans. Our previous studies indicated that the S100A12 gene was abundant in the immune tissues of pigs and was significantly upregulated during infection with *Haemophilus parasuis* (HPS) or porcine circovirus type 2 (PCV2). In this study, the mechanism of transcriptional regulation of S100A12 was investigated in pigs. Our results showed that S100A12, CCAAT/enhancer-binding protein beta (C/EBPβ) and activator protein-1 (AP-1) genes were up-regulated in PK-15 (ATCC, CCL-33) cells when treated with LPS or Poly I: C. Additionally, the promoter activity and expression level of the S100A12 gene were significantly upregulated when C/EBPβ or AP-1 were overexpressed. We utilized electromobility shift assays (EMSA) to confirm that C/EBPβ and AP-1 could directly bind the S100A12 gene promoter. We also found that the transcriptional activity and expression levels of C/EBPβ and AP-1 could positively regulate each other. Furthermore, the promoter activity of the S100A12 gene was higher when C/EBPβ and AP-1 were cotransfected than when they were transfected individually. We concluded that the S100A12 gene was cooperatively and positively regulated by C/EBPβ and AP-1 in pigs. Our study offers new insight into the transcriptional regulation of the S100A12 gene.

## 1. Introduction

The S100 proteins are small, acidic, calcium-binding proteins with “EF-hand type” conformation. To date, at least 25 S100 proteins have been identified in humans [1]. S100A12, also known as CAAF1 and calgranulin C, is a member of the S100 family, which is expressed specifically in mammals, with the exception of mice and rats [2]. According to previous studies, S100A12 is a proinflammatory factor that is up-regulated at sites of inflammation. In patients with inflammatory disorders, the serum concentration of S100A12 is increased. S100A12 is considered an important marker for many inflammatory diseases in humans including arthritis, asthma, cystic fibrosis, and chronic inflammatory bowel diseases [3,4,5,6]. It has been reported that the binding of S100A12 to advanced glycosylation end product-specific receptor (AGER) mediates the important proinflammatory axis [7]. AGER belongs to the immunoglobulin protein family of cell surface molecules. The AGER protein contains an extracellular domain, a single transmembrane spanning helix and a cytosolic domain. The extracellular domain contains one variable V-like domain and two constant C-like domains [8,9]. S100A12 was shown to bind the V-domain to activate the downstream transcription factor NFκB, which mediates the inflammatory response [7]. Recently, one study demonstrated that S100A12 could stimulate secretion of IL-8 and TNF-α via Toll-like receptor 4 (TLR4) in human monocytes and HEK-293 cells [10]. Furthermore, S100A12 could modulate migration and provoke the activation of monocytes and mast cells [6]. The S100A12 gene could also induce the expression of adhesion molecules, such as vascular cell adhesion molecule-1 (VCAM-1) and intercellular adhesion molecule-1 (ICAM-1) [11]. These results indicate that S100A12 plays an important role in inflammatory response.

In addition to its role in the inflammatory response, S100A12 also participates in other biological processes. It was reported that S100A12 plays a role in type 2 diabetes. The plasma concentration of S100A12 in patients with diabetes was two-fold higher than in controls. Additionally, the concentration of S100A12 in the plasma was positively correlated with hemoglobin A1c and blood glucose levels [12]. In primary human aortic endothelial cells, high glucose stimulation increased S100A12 gene expression [13]. Furthermore, investigation of proteins interacting with S100A12 indicated that several metabolic enzymes, including cytosolic NADP+-dependent isocitrate dehydrogenase (IDH), fructose-1,6-bisphosphate aldolase A (Aldolase), glyceraldehyde-3-phosphate dehydrogenese (GAPDH), and annexin V, could interact with S100A12 directly [14]. These studies indicated that S100A12 was involved in diabetes. In addition, overexpression of S100A12 in mouse vascular smooth muscle could mediate aortic wall remodeling and aortic aneurysm [11].

Expression pattern analysis indicated that the S100A12 gene was abundant in granulocytes, detectable in monocytes and lymphocytes, and significantly up-regulated at inflammatory sites in humans [15,16]. In pigs, we found that the S100A12 gene was highly expressed in lymph nodes, bone marrow, skin, and lung tissues, and was detectable in the liver, kidney and fat tissues. Expression of the S100A12 gene was up-regulated in the spleen and blood during infection with *Haemophilus parasuis* (HPS), porcine circovirus type 2 (PCV2), or after stimulation with lipopolysaccharides (LPS) or Poly I: C [17,18]. Investigation of transcriptional regulation indicated that the −135 to −50 region of the promoter of the porcine S100A12 gene was important for transcription [19]. Additionally, the S100A12 gene had a similar expression pattern to the S100A8 and S100A9 genes [17]. In mice, it has been confirmed that S100A8 forms a complex with S100A9 and serves as an endogenous ligand of TLR4 to participate in the inflammatory response [20]. The S100A8/A9/A12 genes may, therefore, also play important roles in the inflammatory response in pigs.

Although the S100A12 gene is important in the inflammatory response, the mechanism of transcriptional regulation of the S100A12 gene is not clear. In this study, transcriptional regulation of the S100A12 gene was investigated in pigs. Our results indicated that transcription of S100A12 was positively regulated by both CCAAT/enhancer-binding protein beta (C/EBPβ) and activator protein-1 (AP-1). In addition, synergetic effects were observed for these two transcription factors in the transcriptional activation of S100A12. The S100A8 and S100A9 genes have similar expression patterns to S100A12 in pigs and contain conserved C/EBPβ and AP-1 binding sites. These genes may, therefore, also be regulated by C/EBPβ and AP-1.

## 2. Results and Discussion

### 2.1. Results

#### 2.1.1. Abolished AP-1 and C/EBPβ Binding Sites Decrease Porcine S100A12 Gene Promoter Activity

**Figure 1:**
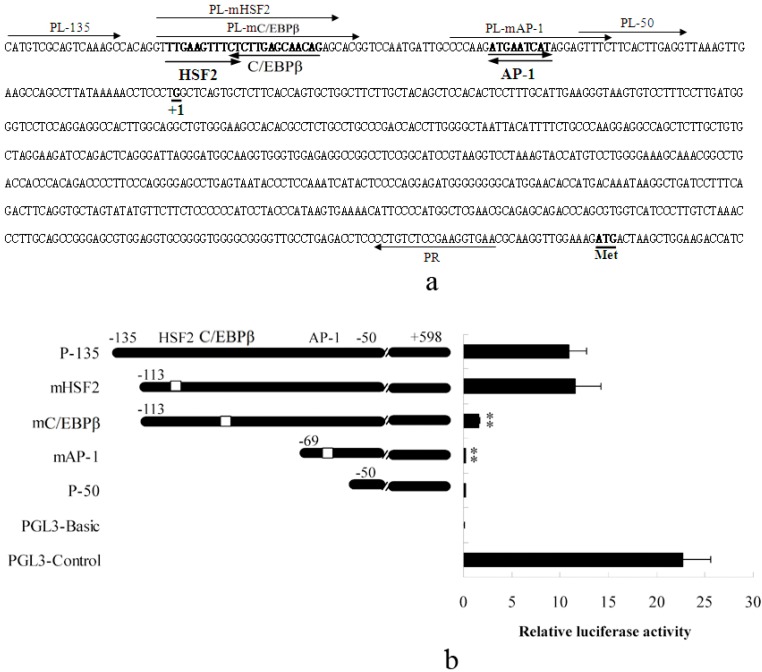
Transcriptional activity of the porcine S100A12 gene promoter. (**a**) Binding sites of the transcription factors HSF2, C/EBPβ, and AP-1 are shown in bold and the orientation of these transcription factors are indicated by bold arrows. The primers for promoter fragment isolation and binding site mutation are also indicated by arrows. +1 indicates the transcription start site; ATG, the initiating codon of the coding region; (**b**) The transcriptional activity of the porcine S100A12 gene promoter was significantly decreased when C/EBPβ binding site was mutated and almost abolished when both C/EBPβ and AP-1 binding sites were deleted. P-135 contains regions −135 to +598 of the promoter; mHSF2 contains regions −113 to +598 with a mutated HSF2 binding site; mC/EBPβ contains regions −113 to +598 with a mutated C/EBPβ binding site; mAP-1 contains regions −69 to +598 with a mutated AP-1 binding site and P-50 contains regions −50 to +598. All fragments were cloned into a pGL3 vector, and the pGL3-Basic and pGL3-Control were used as negative and positive controls, respectively. Error bars represent mean ± SEM, representative of at least three experiments. mAP-1, C/EBPβ Vs P-135; ** indicates significant differences (*p* < 0.01) compared to P-135.

The −135 to −50 region of the porcine S100A12 gene promoter was determined to be important for its transcriptional activation in our previous study [19]. In this study, transcription factor binding site analysis was performed using TFSEARCH tool and HSF2 (Heat shock transcription factor 2), C/EBPβ and AP-1 binding sites were identified in this region (Figure 1a). Luciferase reporter gene analysis results indicated that the activity of the P-135 fragment was significantly higher than P-50 fragment, which was consistent with our previous study [19]. Furthermore, mutation of the HSF2 binding site (mHSF2) did not affect the transcriptional activity. Mutation of C/EBPβ binding site (mC/EBPβ) significantly decreased transcriptional activity. Mutation of AP-1 binding site together with deletion of the C/EBPβ site (mAP-1) nearly abolished transcriptional activity entirely (Figure 1b). These results indicated that transcription of the porcine S100A12 gene was positively regulated by C/EBPβ and AP-1.

#### 2.1.2. Promoter Activity and Expression of the S100A12 Gene Were Increased When AP-1 or C/EBPβ Were Over-Expressed

**Figure 2:**
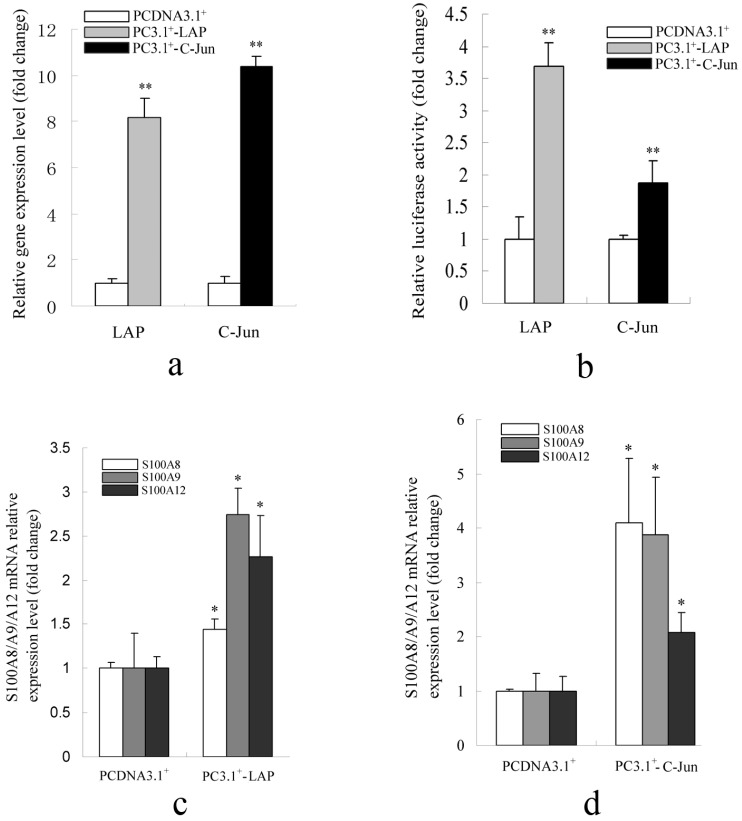
Promoter activity and expression pattern of S100A12 gene when LAP or C-Jun were overexpressed. (**a**) The detection of the expression level of C/EBPβ and C-Jun when their overexpression constructions were transfected in PK-15 cells; (**b**) The promoter activity of S100A12 was increased when LAP or C-Jun were overexpressed; (**c**) The mRNA levels of S100A8/A9/A12 were increased when LAP was overexpressed; (**d**) The mRNA levels of S100A8/A9/A12 were increased when C-Jun was overexpressed. PCDNA3.1^+^ was used as negative control. The value of the PCDNA3.1^+^ (control group) was set as 1. Error bars represent mean ± SEM, representative of at least three experiments. LAP, C-Jun *vs.* PCDNA3.1^+^; * indicates a significant difference (*p* < 0.05); ** indicates a significant difference (*p* < 0.01).

The expression of LAP or C-Jun was significantly increased when their overexpression constructs were transfected in PK-15 (ATCC, CCL-33) cells (Figure 2a). Luciferase assay results indicated that overexpression of LAP and C-Jun significantly increased transcriptional activation of the −135 to +598 region of porcine S100A12 gene (Figure 2b). LAP is the active isoform of C/EBPβ and *C*-*Jun* is a transcriptional activation subunit of AP-1. These results also suggest that C/EBPβ and AP-1 positively regulated transcription of the porcine S100A12 gene. Furthermore, the mRNA levels of S100A8/A9/A12 were significantly up-regulated when LAP or C-Jun were overexpressed (Figure 2c,d). These results confirmed that the porcine S100A12 gene is positively regulated by C/EBPβ and AP-1. C/EBPβ and AP-1 binding sites were also identified in the −1200 bp promoter region of the porcine S100A8 and S100A9 genes. The positioning of some binding sites was conserved in pigs and humans (supplementary Figure 1). The S100A8 and S100A9 genes may, therefore, also be regulated by these two transcription factors.

#### 2.1.3. S100A8/A9/A12, AP-1 and C/EBPβ Genes Were Up-Regulated in PK-15 Cells When Treated with LPS or Poly I: C

**Figure 3:**
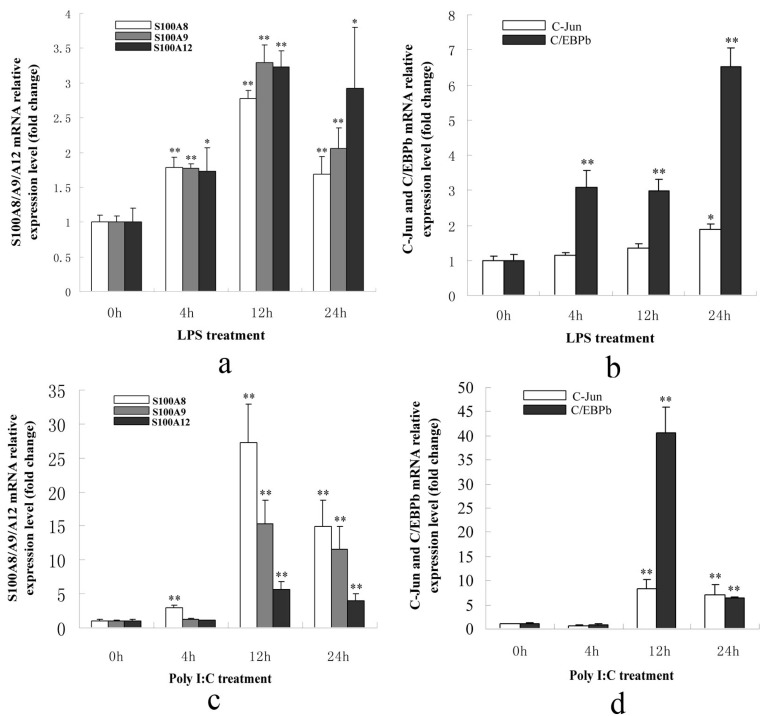
The expression patterns of porcine S100A8/A9/A12, C/EBPβ and C-Jun genes in PK-15 cells after treatment with LPS or Poly I: C. (**a**) The expression of S100A8/A9/A12 genes was significantly up-regulated after stimulation with LPS for 4, 12, and 24 h; (**b**) The expression of C/EBPβ was significantly up-regulated after stimulation with LPS for 4, 12, and 24 h. The C-Jun was significantly up-regulated at 24 h following LPS stimulation; (**c**) The expression of S100A8/A9/A12 was significantly up-regulated after stimulation with Poly I: C for 12 and 24 h; (**d**) The expression of C/EBPβ and C-Jun genes was significantly up-regulated after stimulation with Poly I: C for 12 and 24 h. The expression of genes at 0 h was set as 1. Error bars represent mean ± SEM, representative of at least three experiments. 4, 12, 24 h *vs.* 0 h; * indicates significant differences (*p* < 0.05); ** indicates significant differences (*p* < 0.01).

Total RNA was extracted from PK-15 cells after treatment with 1 μg/mL LPS or 10 μg/mL Poly I: C for 0, 4, 12, or 24 h. Q-PCR was performed and the results indicated that the S100A8/A9/A12 genes were significantly up-regulated at 12 and 24 h post-treatment with LPS or Poly I: C. The AP-1 and C/EBPβ genes were significantly up-regulated after 12 and 24 h of treatment. Therefore, these five genes were up-regulated under the stimulation of LPS or Poly I: C (Figure 3a–d). They also indicated that S100A8/A9/A12 genes could be regulated by AP-1 and C/EBPβ. Moreover, the fold change in C/EBPβ expression was much higher than for C-Jun after 12 h of Poly I: C treatment, and after 4, 12, and 24 h of LPS treatment. These results indicated that changes in the transcriptional activity of C/EBPβ were likely due to increased expression levels when stimulated with LPS or Poly I: C.

#### 2.1.4. C/EBPβ and AP-1 Directly Bind the Promoter of the Porcine S100A12 Gene

**Figure 4:**
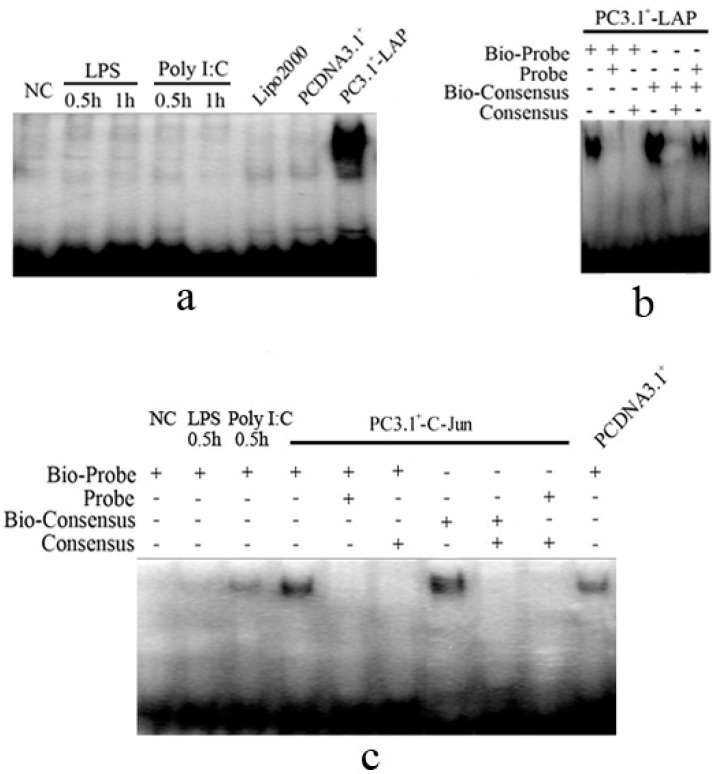
EMSA results for C/EBPβ and AP-1 binding. (**a**) The EMSA of C/EBPβ. The binding ability of C/EBPβ was increased sharply when LAP was overexpressed and it was not altered after stimulation with LPS or Poly I: C. Lipo2000 indicates PK-15 cells treated only with Lipofectamine™ 2000, which was used as a negative control; (**b**) The EMSA of C/EBPβ containing the unlabeled competitive probe. The binding of LAP could be competitively inhibited by 100-fold excess unlabeled competitive C/EBPβ probe of S100A12 or by 100-fold excess unlabeled consensus probe of C/EBPβ. Bio-probe indicates a C/EBPβ probe of S100A12 labeled with biotin; probe indicates a C/EBPβ probe of the porcine S100A12 gene without biotin labeling, which was used in a competition reaction; Bio-consensus indicates the consensus probe of C/EBPβ labeled by biotin; Consensus indicates the consensus probe of C/EBPβ without biotin labeling, which was used as competition reaction; (**c**) The EMSA of AP-1. The binding ability of AP-1 was increased obviously when C-Jun was overexpressed compare to PCDNA3.1^+^ group and it was slightly increased when treated with Poly I: C or LPS for 30 min compare to the NC group. The binding ability of AP-1 could be inhibited by 100-fold excess unlabeled competitive probe of S100A12 or by 100-fold unlabeled competitive consensus probe of AP-1. Bio-probe indicates the AP-1 probe of the porcine S100A12 gene labeled with biotin; probe indicates the AP-1 probe of porcine S100A12 gene without biotin labeling, which was used in competition reactions; Bio-consensus indicates the consensus probe of AP-1 labeled by biotin; consensus indicates the consensus probe of AP-1 without biotin labeling, which was used in competition reactions; NC indicates PK-15 cells without any treatment, which were used as negative control for the treatments of LPS and Poly I: C; PCDNA3.1^+^ indicates PK-15 cells transfected with the PCDNA3.1^+^ vector, which was used as negative control for the overexpression of C-Jun; PC3.1^+^-LAP and PC3.1^+^-C-Jun indicate that LAP and C-Jun were overexpressed, respectively.

PK-15 cells were treated with LPS, Poly I: C or overexpressed with LAP and C-Jun. Nuclear proteins were extracted for electromobility shift assay (EMSA) analysis. The results showed that binding of C/EBPβ to the S100A12 C/EBPβ probe did not occur after stimulation with LPS or Poly I: C for 30 min or 1 h. Over expression of LAP enhanced binding C/EBPβ (Figure 4a). Binding of C/EBPβ to the S100A12 C/EBPβ probe could be completely abolished in the presence of 100-fold excess of unlabeled S100A12 C/EBPβ probe or 100-fold excess unlabeled consensus probe of C/EBPβ. The binding of C/EBPβ to its consensus probe could be competitively inhibited by 100-fold excess of unlabeled C/EBPβ consensus probe, as well as by 100-fold excess of unlabeled S100A12 C/EBPβ probe (Figure 4b). The binding of AP-1 to the S100A12 AP-1 probe was also enhanced when C-Jun was over expressed compare to the negative control of PCDNA3.1^+^. Binding could be competitively inhibited by 100-fold excess S100A12 AP-1 probe or by 100-fold excess of unlabeled AP-1 consensus probe. In addition, binding of AP-1 to the S100A12 AP-1 probe could be enhanced slightly by stimulation with Poly I: C or LPS compare to the NC group (Negative control, PK-15 cells without stimulation) (Figure 4C). These results indicated that AP-1 could be rapidly activated after stimulation with Poly I: C and LPS for the transcriptional regulation of the porcine S100A12 gene. In contrast, the binding of C/EBPβ was not altered by these stimulations.

#### 2.1.5. C/EBPβ and AP-1 Act Synergistically in the Transcriptional Activation of the Porcine S100A12 Gene

**Figure 5:**
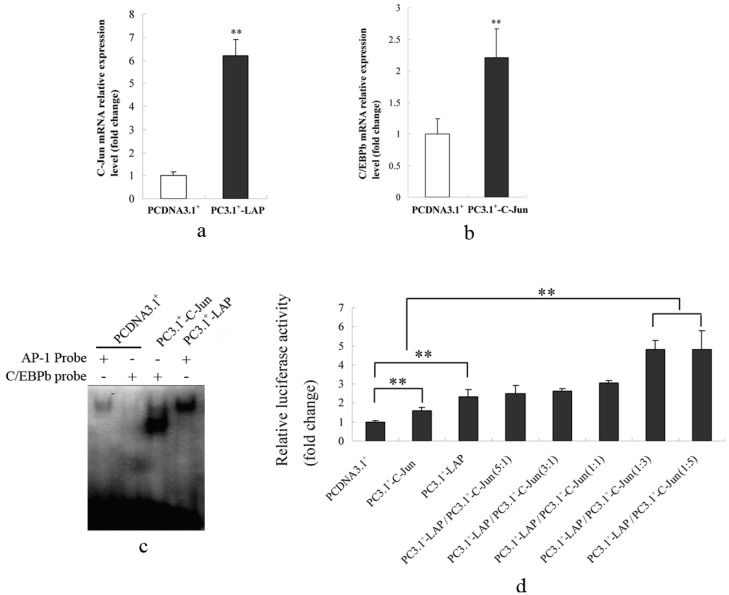
Synergetic effects of C/EBPβ and AP-1 in the transcriptional regulation of the porcine S100A12 gene in PK-15 cells. (**a**) C/EBPβ transcription levels were significantly increased when C-Jun was overexpressed; (**b**) C-Jun transcription levels were significantly increased when LAP was overexpressed; (**c**) Nuclear proteins were extracted for EMSA 24 h following transfection of PC3.1^+^-LAP or PC3.1^+^-C-Jun. C/EBPβ binding ability was increased when C-Jun was overexpressed. AP-1 binding ability was also increased when LAP was overexpressed; (**d**) Over expression constructs of LAP and C-Jun were transfected into PK-15 cells together at different ratio of 5:1, 3:1, 1:1, 1:3, and 1:5, or LAP, and C-Jun were transfected alone. Transcriptional activity of the promoter was then studied using luciferase reporter gene. The promoter activity after cotransfection of constructs of LAP and C-Jun at 1:3 or 1:5 was significant higher than when constructs of LAP or C-Jun were transfected alone. PCDNA3.1^+^ vector was used as the negative control and its value was set as 1. Error bars represent mean ± SEM, representative of at least three experiments. ** indicates a significant difference (*p* < 0.01).

Q-PCR results showed that C/EBPβ mRNA was significantly up-regulated when C-Jun was overexpressed. Additionally, C-Jun mRNA was up-regulated approximately six-fold when LAP was overexpressed (Figure 5a,b). These results indicated that C/EBPβ and AP-1 could regulate each other. EMSA results also indicated that the C/EBPβ binding ability increased sharply when C-Jun was overexpressed and AP-1 binding ability also increased when LAP was overexpressed (Figure 5c). Moreover, the transcriptional activity of the promoter of porcine S100A12 gene was increased significantly more when overexpression constructs of LAP and C-Jun were transfected together at ratio of 1:3 or 1:5 than when overexpression constructs of C-Jun or LAP were transfected alone (Figure 5d). These results indicated that C/EBPβ and AP-1 acted synergistically in the transcriptional activation of the porcine S100A12 gene.

### 2.2. Discussion

The S100A12 gene belongs to the S100 gene family, which plays very important roles in the inflammatory response. In humans, it is considered a marker for many types of inflammatory disease including arthritis, asthma, cystic fibrosis and chronic inflammatory bowel diseases [3,4,5,6]. In pigs, the S100A12 gene is highly expressed in immune-related tissues and is up-regulated during infections with HPS, *Salmonella*, or PCV2 [17,19,21]. Expression of the S100A12 gene was up-regulated after stimulation with LPS or Poly I: C indicating that S100A12 is likely also important in the inflammatory response in pigs [19]. In our previous study, we found that the −135 to −50 promoter region of porcine S100A12 gene was related to its transcriptional activation [19]. In this study, transcriptional regulation of this region was studied in greater detail. Based on our results, the transcriptional activity of the porcine S100A12 gene promoter was decreased significantly when C/EBPβ or AP-1 binding sites were mutated. C/EBPβ and AP-1 could directly bind the promoter and upregulate expression of the porcine S100A12 gene. These results confirmed that the porcine S100A12 gene is positively transcriptionally regulated by C/EBPβ and AP-1. We also compared the expression patterns and the promoter structures of the S100A8/A9/A12 genes and found that they were quite similar. We hypothesize that transcriptional regulation of S100A8/A9 may occur by an identical mechanism as S100A12 and that transcription of S100A8/A9 could be positively regulated by C/EBPβ and AP-1 in pigs. In humans, one study suggested involvement of AP-1 in transcription of the S100A12/A8 genes after using ChIP assays in aortic endothelial cells to confirm that AP-1 could bind to the promoters of S100A12 and S100A8 [13]. The transcriptional regulation of S100A8/A12 is, therefore, likely to be similar in humans and pigs.

In this study, synergetic effects between C/EBPβ and AP-1 were found in the transcriptional regulation of the S100A12 gene. We found that there was reciprocal regulation between C/EBPβ and AP-1. Additionally, the promoter activity of the porcine S100A12 gene was higher when C/EBPβ and AP-1 were cotransfected than when C/EBPβ or AP-1 were transfected alone. Bioinformatics analysis identified an AP-1 binding site at approximately −800 bp of the promoter of the porcine C/EBPβ gene, which was conserved across pigs, humans and mice. Similarly, there were several C/EBPβ binding sites at −1000 to +1 bp of the promoter region of the C-Jun gene (data not shown). We hypothesized that the synergetic effects of C/EBPβ and AP-1 may also exist in other species. Previous studies reported that C/EBPβ had synergetic effects with NFκB in human Hep 3B cells and in rat hepatocytes [22,23]. Here, we offered new evidence of the synergetic effects of C/EBPβ and AP-1 in pigs.

It has been reported that C/EBPβ plays important roles in the immune response. C/EBPβ induces transcription of important immune-related genes including IL-6, mcp-1, MIP-1α, MIP-1β, osteopontin, CD14, and lysozyme [24,25,26]. In addition, peritoneal macrophages from C/EBPβ deficient mice were defective in intracellular killing, and mutant mice were highly susceptible to infections with microorganisms such as *Listeria monocytogenes*, *Salmonella typhimurium*, and *Candida albicans* [27,28]. C/EBPβ has been demonstrated to have three isoforms; 38 kDa (LAP_f_), 34 kDa (LAP), and 20 kDa (LIP), which are translated from one transcript in mice. LAP is the main active isoform and its protein levels are much higher than the other two isoforms [29]. In this study, the LAP was over exprssed. As expected, LAP increased promoter activity and the expression of the porcine S100A12 gene.

AP-1 also participates in the immune response. It is activated by the *TLR* signaling pathway [30]. AP-1 can induce expression of IL-6, IL-8, IL-4 and MHC genes [31,32,33,34,35]. The AP-1 family is a family of dimeric complexes of Jun (C-Jun, JunB, and JunD) homodimers and Jun heterodimers with Fos (c-Fos, FosB, Fra-1, and Fra-2) [36,37]. They share a conserved bZIP domain, which can bind to TPA-responsive elements (TREs) containing the consensus motif 5'-TGAG/CTCA-3' [38]. It was reported that C-Jun is the transcriptional activation unit and overexpression of C-Jun alone is sufficient for the activation of AP-1 [39,40]. In this study, we found that AP-1 binding ability was significantly enhanced when C-Jun was over expressed, and that C-Jun over-expression alone is sufficient to activate the expression of the porcine S100A12 gene.

## 3. Materials and Methods

### 3.1. Cells and Vectors

In this study, PK-15 (ATCC, CCL 32) cells, which was derived form pig kidney, were cultured in Dulbecco’s Modified Eagle’s Medium supplemented with 10% fetal bovine serum at 37 °C and 5% CO_2_. For LPS or Poly I: C treatment, approximately 1 × 10^5^ cells were cultured in 6-well plate for 24 h. Fresh medium with 1 μg/mL LPS (*E. coli* 0127:B8, Sigma, St. Louis, MO, USA) or 10 μg/mL Poly I: C (Sigma, St. Louis, MO, USA) was then added. PK-15 cells were harvested at 0, 4, 12, or 24 h after LPS or Poly I: C treatment.

The expression vector PCDNA3.1^+^ (Invitrogen, Carlsbad, CA, USA) was used for over expression of genes. For promoter activity analysis, the pGL3 and pRL-TK (Promega, Madison, WI, USA) vectors were used. The vectors used for overexpression of C-Jun (AP-1 transcriptional active subunit) and LAP (active form of C/EBPβ) were designated PC3.1^+^-C-Jun and PC3.1^+^-LAP, respectively. A series of fragments of the porcine S100A12 gene promoter were cloned into the pGL3 vector for transcriptional activity analysis, and were designated P-135 (contains regions −135 to +598 of the promoter of porcine S100A12), mHSF2 (contains regions −113 to +598 and a mutated HSF2 binding site), *m*C/EBPβ (contains regions −113 to +598 and the mutant C/EBPβ binding site), *m*AP-1 (contains regions −69 to +598 and a mutated AP-1 binding site) and P-50 (contains regions −50 to +598). The primers used for vector construction are listed in supplementary Table 1. All these constructs were confirmed by sequence. Significance was analyzed using the *t*-test.

### 3.2. Quantitative Polymerase Chain Reaction

For quantitative polymerase chain reaction (Q-PCR) analysis, total cellular RNA was extracted using TRIzol reagent (Invitrogen, Carlsbad, CA, USA). Reverse transcription was performed using the TransSript First-Strand cDNA Synthesis SuperMix kit according to the manufacturer’s instructions (Tiangen, Beijing, China). Q-PCR was performed using a standard SYBR Green PCR kit (Toyobo, Osaka, Japan) and the Lightcycle 480 PCR machine (Roche, Penzberg, Germany). The PCR conditions were: denaturation at 95 °C for 3 min, 95 °C for 30 s, annealing at 72 °C for 30 s, and extension at 72 °C for 30 s for a total of 40 cycles. Melting curves were obtained by increasing the temperature from 55 to 95 °C at a rate of 0.5 °C/s for 10 s. The *RPL32* gene was used as an internal control. Primers used for Q-PCR detection are listed in supplementary Table 1.

### 3.3. Luciferase Reporter Gene Analysis

For analysis of promoter activity of the porcine S100A12 gene, PK-15 cells in 24-well plates were transfected with the addition of 50 μL Opti-MEM I medium (Invitrogen, Carlsbad, CA, USA), containing 2 μL Lipofectamine 2000 reagent (Invitrogen, Carlsbad, CA, USA), 0.072 μg of pRL-TK (Promega, Madison, WI, USA) and 0.72 μg of each construct of P-135, mHSF2, mC/EBPβ, mAP-1 or P-50. pGL3-Basic and pGL3-Control vectors (Promega, Madison, WI, USA) were used as the negative and positive controls, respectively. For investigation of C/EBPβ and AP-1 synergy, PK-15 cells were transfected with PCDNA3.1^+^, PC3.1^+^ C-Jun, PC3.1^+^ LAP at various ratios of PC3.1^+^ C-Jun and PC3.1^+^ LAP including 5:1, 3:1, 1:1, 1:3 and 1:5. The total quantity of PC3.1^+^ C-Jun and PC3.1^+^ LAP in each group was 0.36 μg. *P-135* (0.36 μg) and *pRL-TK* (0.036 μg) were also cotransfected. Cell lysates were collected 24 h after transfection. Firefly luciferase and Renilla luciferase activity was measured using the Dual-Luciferase Reporter kit (Promega, Madison, WI, USA) and the VICTOR ×2 system (PerkinElmer, Waltham, MA, USA). The activity of the promoter was determined using the ratio of Firefly luciferase/Renilla luciferase activity.

### 3.4. Electromobility Shift Assay (EMSA) for Detection of DNA Binding Abilities of C/EBPβ and AP-1

PK-15 cells in 6-well plates were treated with 1 μg/mL LPS or 10 μg/mL Poly I: C for 30 min or 1 h. PK-15 cells were transfected with PC3.1^+^-C-Jun and PC3.1^+^-LAP constructs. At 12 h post-transfection, nuclear proteins from each group were extracted using a nuclear protein extraction kit following the manufacturer’s instructions (Viagene Biotech, Changzhou, China). Equal amounts of nuclear protein extract (2 μg) from each treatment were incubated with a 15 μL mixture containing 1.5 μL 10× binding buffer, l μg Poly(dI-dC) and 8.3 nM biotin-labeled C/EBPβ or AP-1 probe. The binding reaction was performed for 25 min at room temperature. The DNA–protein complexes were electrophoresed on 6.5% non-denaturing polyacrylamide gels. Electrotransfer and biotin label detection were performed according to the kit protocols (Viagene Biotech, Changzhou, China). The probes used in the EMSA are listed in supplementary Table 1.

## 4. Conclusions

In conclusion, we investigated the mechanism of transcriptional regulation of the S100A12 gene in pigs. Our results indicated that S100A12 was positively regulated by C/EBPβ and AP-1 and that these two transcriptional factors had synergetic effects in the transcriptional activation of the S100A12 gene. The porcine S100A8 and S100A9 genes may also be regulated by these transcription factors.

## Supplementary Files

Supplementary File 1

## References

[1] Santamaria-Kisiel L., Rintala-Dempsey A.C., Shaw G.S. (2006). Calcium-dependent and -independent interactions of the S100 protein family. Biochem. J..

[2] Ravasi T., Hsu K., Goyette J., Schroder K., Yang Z., Rahimi F., Miranda L.P., Alewood P.F., Hume D.A., Geczy C. (2004). Probing the S100 protein family through genomic and functional analysis. Genomics.

[3] Foell D., Frosch M., Sorg C., Roth J. (2004). Phagocyte-specific calcium-binding S100 proteins as clinical laboratory markers of inflammation. Clin. Chim. Acta.

[4] Foell D., Seeliger S., Vogl T., Koch H.G., Maschek H., Harms E., Sorg C., Roth J. (2003). Expression of S100A12 (EN-RAGE) in cystic fibrosis. Thorax.

[5] Yang Z., Tao T., Raftery M.J., Youssef P., di Girolamo N., Geczy C.L. (2001). Proinflammatory properties of the human S100 protein S100A12. J. Leukoc. Biol..

[6] Yang Z., Yan W.X., Cai H., Tedla N., Armishaw C., di Girolamo N., Wang H.W., Hampartzoumian T., Simpson J.L., Gibson P.G. (2007). S100A12 provokes mast cell activation: A potential amplification pathway in asthma and innate immunity. J. Allergy Clin. Immunol..

[7] Hofmann M.A., Drury S., Fu C., Qu W., Taguchi A., Lu Y., Avila C., Kambham N., Bierhaus A., Nawroth P. (1999). RAGE mediates a novel proinflammatory axis: A central cell surface receptor for S100/calgranulin polypeptides. Cell.

[8] Neeper M., Schmidt A.M., Brett J., Yan S.D., Wang F., Pan Y.C., Elliston K., Stern D., Shaw A. (1992). Cloning and expression of a cell surface receptor for advanced glycosylation end products of proteins. J. Biol. Chem..

[9] Srikrishna G., Huttunen H.J., Johansson L., Weigle B., Yamaguchi Y., Rauvala H., Freeze H.H. (2002). *N*-Glycans on the receptor for advanced glycation end products influence amphoterin binding and neurite outgrowth. J. Neurochem..

[10] Foell D., Wittkowski H., Kessel C., Luken A., Weinhage T., Varga G., Vogl T., Wirth T., Viemann D., Bjork P. (2013). Proinflammatory S100A12 can activate human monocytes *via* Toll-like receptor 4. Am. J. Respir. Crit. Care Med..

[11] Hofmann B.M., Wilk J., Heydemann A., Kim G., Rehman J., Lodato J.A., Raman J., McNally E.M. (2010). S100A12 mediates aortic wall remodeling and aortic aneurysm. Circ. Res..

[12] Kosaki A., Hasegawa T., Kimura T., Iida K., Hitomi J., Matsubara H., Mori Y., Okigaki M., Toyoda N., Masaki H. (2004). Increased plasma S100A12 (EN-RAGE) levels in patients with type 2 diabetes. J. Clin. Endocrinol. Metab..

[13] Yao D., Brownlee M. (2010). Hyperglycemia-induced reactive oxygen species increase expression of the receptor for advanced glycation end products (RAGE) and RAGE ligands. Diabetes.

[14] Hatakeyama T., Okada M., Shimamoto S., Kubota Y., Kobayashi R. (2004). Identification of intracellular target proteins of the calcium-signaling protein S100A12. Eur. J. Biochem..

[15] Dell’Angelica E.C., Schleicher C.H., Santome J.A. (1994). Primary structure and binding properties of calgranulin C, a novel S100-like calcium-binding protein from pig granulocytes. J. Biol. Chem..

[16] Guignard F., Mauel J., Markert M. (1995). Identification and characterization of a novel human neutrophil protein related to the S100 family. Biochem. J..

[17] Chen H., Li C., Fang M., Zhu M., Li X., Zhou R., Li K., Zhao S. (2009). Understanding *Haemophilus parasuis* infection in porcine spleen through a transcriptomics approach. BMC Genomics.

[18] Chen H., Lunney J.K., Cheng L., Li X., Cao J., Zhu M., Zhao S. (2011). Porcine S100A8 and S100A9: Molecular characterizations and crucial functions in response to *Haemophilus parasuis* infection. Dev. Comp. Immunol..

[19] Chen H., Cheng L., Yang S., Liu X., Liu Y., Tang J., Li X., He Q., Zhao S. (2010). Molecular characterization, induced expression, and transcriptional regulation of porcine S100A12 gene. Mol. Immunol..

[20] Vogl T., Tenbrock K., Ludwig S., Leukert N., Ehrhardt C., van Zoelen M.A., Nacken W., Foell D., van der Poll T., Sorg C. (2007). Mrp8 and Mrp14 are endogenous activators of Toll-like receptor 4, promoting lethal, endotoxin-induced shock. Nat. Med..

[21] Wang Y., Qu L., Uthe J.J., Bearson S.M., Kuhar D., Lunney J.K., Couture O.P., Nettleton D., Dekkers J.C., Tuggle C.K. (2007). Global transcriptional response of porcine mesenteric lymph nodes to *Salmonella enterica* serovar Typhimurium. Genomics.

[22] Sakitani K., Nishizawa M., Inoue K., Masu Y., Okumura T., Ito S. (1998). Synergistic regulation of inducible nitric oxide synthase gene by CCAAT/enhancer-binding protein beta and nuclear factor-kappaB in hepatocytes. Genes Cells.

[23] Agrawal A., Cha-Molstad H., Samols D., Kushner I. (2001). Transactivation of C-reactive protein by IL-6 requires synergistic interaction of CCAAT/enhancer binding protein beta (C/EBP beta) and Rel p50. J. Immunol..

[24] Bretz J.D., Williams S.C., Baer M., Johnson P.F., Schwartz R.C. (1994). C/EBP-related protein 2 confers lipopolysaccharide-inducible expression of interleukin 6 and monocyte chemoattractant protein 1 to a lymphoblastic cell line. Proc. Natl. Acad. Sci. USA.

[25] Matsumoto M., Sakao Y., Akira S. (1998). Inducible expression of nuclear factor IL-6 increases endogenous gene expression of macrophage inflammatory protein-1 alpha, osteopontin and CD14 in a monocytic leukemia cell line. Int. Immunol..

[26] Uematsu S., Kaisho T., Tanaka T., Matsumoto M., Yamakami M., Omori H., Yamamoto M., Yoshimori T., Akira S. (2007). The C/EBP beta isoform 34-kDa LAP is responsible for NF-IL-6-mediated gene induction in activated macrophages, but is not essential for intracellular bacteria killing. J. Immunol..

[27] Tanaka T., Akira S., Yoshida K., Umemoto M., Yoneda Y., Shirafuji N., Fujiwara H., Suematsu S., Yoshida N., Kishimoto T. (1995). Targeted disruption of the *NF-IL6* gene discloses its essential role in bacteria killing and tumor cytotoxicity by macrophages. Cell.

[28] Screpanti I., Romani L., Musiani P., Modesti A., Fattori E., Lazzaro D., Sellitto C., Scarpa S., Bellavia D., Lattanzio G. (1995). Lymphoproliferative disorder and imbalanced T-helper response in C/EBP beta-deficient mice. EMBO J..

[29] Descombes P., Schibler U. (1991). A liver-enriched transcriptional activator protein, LAP, and a transcriptional inhibitory protein, LIP, are translated from the same mRNA. Cell.

[30] Liu W., Ouyang X., Yang J., Liu J., Li Q., Gu Y., Fukata M., Lin T., He J.C., Abreu M. (2009). AP-1 activated by toll-like receptors regulates expression of IL-23 p19. J. Biol. Chem..

[31] Rohrbach S., Engelhardt S., Lohse M.J., Werdan K., Holtz J., Muller-Werdan U. (2007). Activation of AP-1 contributes to the beta-adrenoceptor-mediated myocardial induction of interleukin-6. Mol. Med..

[32] Kang S.S., Woo S.S., Im J., Yang J.S., Yun C.H., Ju H.R., Son C.G., Moon E.Y., Han S.H. (2007). Human placenta promotes IL-8 expression through activation of JNK/SAPK and transcription factors NF-kappaB and AP-1 in PMA-differentiated THP-1 cells. Int. Immunopharmacol..

[33] Casals C., Barrachina M., Serra M., Lloberas J., Celada A. (2007). Lipopolysaccharide up-regulates MHC class II expression on dendritic cells through an AP-1 enhancer without affecting the levels of CIITA. J. Immunol..

[34] Vanden Bush T.J., Bishop G.A. (2008). TLR7 and CD40 cooperate in IL-6 production *via* enhanced JNK and AP-1 activation. Eur. J. Immunol..

[35] Park J., Chung S.W., Kim S.H., Kim T.S. (2006). Up-regulation of interleukin-4 production via NF-AT/AP-1 activation in T cells by biochanin A, a phytoestrogen and its metabolites. Toxicol. Appl. Pharmacol..

[36] Shaulian E., Karin M. (2002). AP-1 as a regulator of cell life and death. Nat. Cell Biol..

[37] Hess J., Angel P., Schorpp-Kistner M. (2004). AP-1 subunits: Quarrel and harmony among siblings. J. Cell Sci..

[38] Angel P., Karin M. (1991). The role of *Jun*, *Fos* and the AP-1 complex in cell-proliferation and transformation. Biochim. Biophys. Acta.

[39] Yuan H., Pan Y., Young C.Y. (2004). Overexpression of c-Jun induced by quercetin and resverol inhibits the expression and function of the androgen receptor in human prostate cancer cells. Cancer Lett..

[40] Duan L., Sterba K., Kolomeichuk S., Kim H., Brown P.H., Chambers T.C. (2007). Inducible overexpression of C-Jun in MCF7 cells causes resistance to vinblastine via inhibition of drug-induced apoptosis and senescence at a step subsequent to mitotic arrest. Biochem. Pharmacol..

