# Supplementary material for: The Inflammation-Related Gene S100A12 Is Positively Regulated by C/EBPβ and AP-1 in Pigs
Source: Int J Mol Sci. 2014 Aug 8;15(8):13802–16. doi: 10.3390/ijms150813802 (PMC4159825; doi:10.3390/ijms150813802)
Supplement: Supplementary File 1 [file ijms-15-13802-s001.pdf]

Supplementary Information

Figure S1. The C/EBPβ and AP-1 binding sites in the -1200 bp region of the promoter of S100A8/A9/A12 genes in humans and pigs. A blue arrow represents the C/EBPβ binding site. A red arrow represents the AP-1 binding site. The orientation of the arrow represents the direction of transcription factors. The double arrow indicates the transcription factors can bind in both positive and negative directions. TSS, the transcriptional start site; Intron I, the first intron of S100A8/A9/A12 genes; ATG, initiating codon of the gene.

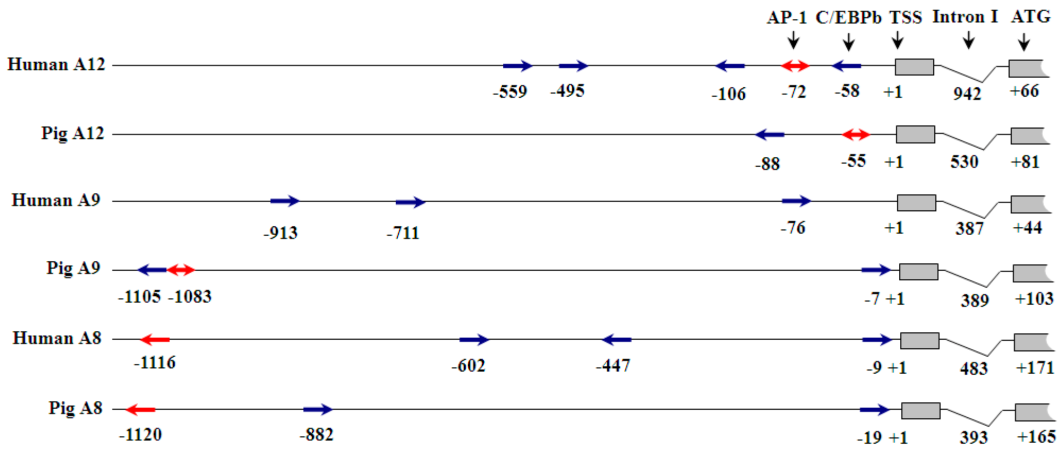

Table 1. The primers and probes used in this study.

Purpose	Name	Sequence (5'–3')
Overexpression vector construction	AP-1L	GTCAAGCTTGACGGACTGTTCTATGACTGC
	AP-1R	GTCGAATTCCTCTCTCTCGCAACTTGTC
	C/EBPb-LAPL	GTCAAGCTTGCCACCATGGAAGTGGCCAACTTC
	C/EBPb-LAPR	GTCGAATTCTAGCAGTGGCCGGAGGA
	C/EBPb-LIPL	GTCAAGCTTGCCACCATGGCGGCTGGCTTCC
	C/EBPb-LIPR	GTCGAATTCTAGCAGTGGCCGGAGGA
Luciferase analysis	PL-135	AGACTCGAGCATGTCGCAGTCAAAGC
	PL-mHSF2	AGACTCGAGGTTTGAAGTCCCTCTTGAGCAACAGAGC
	PL-mC/EBPb	AGACTCGAGGTTTGAAGTTTCTCTTGAGCACGAGAGCAC
	PL-mAP-1	AGACTCGAGCCCCAAGATACATCATAGGAGTTTC
	PL-50	ATCCTCGAGGTTTCTTCACTTGAGG
	PR	AACAAGCTTTTCACCTTCGGAGACAGG
Q-PCR	QAP-1L	TCCAGTAACGGGCACATCAC
	QAP-1R	CCCTCCTGCTCGTCAGTCAC
	QC/EBPL	GTCCACATCCTCGTCGTCCAG
	QC/EBPR	GTTGCGCATCTTGGCCTTGTC
	QS100A8L	TGCTGACGGATCTGGAGAGT
	QS100A8R	CCCACCTTGATCACCAGTATG
	QS100A9L	CGGACCAAATGTCGCAGA
	QS100A9R	CCAGGATGTGGTTTATGGCTTTC
	QS100A12L	GGCATTATGACACCCTTATC
	QS100A12R	GTCACCAGGACCACGAAT
	QRPL32L	CGGAAGTTTCTGGTACACAATGTAA
	QRPL32R	GAAGAGACGTTGTGAGCAA

Table 1. *Cont.*

Purpose	Name	Sequence (5'–3')
EMSA analysis	Consensus C/EBPβ probe	ACCTGCAGATTGCGCAATCTGCACTC
	S100A12 C/EBPβ probe	AAGTTTCTCTTGAGCAACAGAGCACGGT
	Consensus AP-1 probe	CGCTTGATGACTCAGCCGGAA
	S100A12 AP-1 probe	TTGCCCCAAGATGAATCATAGGAGTTTC
